# Size effect, critical resolved shear stress, stacking fault energy, and solid solution strengthening in the CrMnFeCoNi high-entropy alloy

**DOI:** 10.1038/srep35863

**Published:** 2016-10-24

**Authors:** Norihiko L. Okamoto, Shu Fujimoto, Yuki Kambara, Marino Kawamura, Zhenghao M. T. Chen, Hirotaka Matsunoshita, Katsushi Tanaka, Haruyuki Inui, Easo P. George

**Affiliations:** 1 Department of Materials Science and Engineering, Kyoto University, Kyoto 606-8501, Japan; 2 Center for Elements Strategy Initiative for Structure Materials (ESISM), Kyoto University, Kyoto 606-8501, Japan; 3 Department of Mechanical Engineering, Kobe University, Nada-ku, Kobe 657-8501, Japan; 4 Materials Science and Technology Division, Oak Ridge National Laboratory, Oak Ridge, TN 37831, USA

## Abstract

High-entropy alloys (HEAs) comprise a novel class of scientifically and technologically interesting materials. Among these, equatomic CrMnFeCoNi with the face-centered cubic (FCC) structure is noteworthy because its ductility and strength increase with decreasing temperature while maintaining outstanding fracture toughness at cryogenic temperatures. Here we report for the first time by single-crystal micropillar compression that its bulk room temperature critical resolved shear stress (CRSS) is ~33–43 MPa, ~10 times higher than that of pure nickel. CRSS depends on pillar size with an inverse power-law scaling exponent of –0.63 independent of orientation. Planar ½ < 110 > {111} dislocations dissociate into Shockley partials whose separations range from ~3.5–4.5 nm near the screw orientation to ~5–8 nm near the edge, yielding a stacking fault energy of 30 ± 5 mJ/m^2^. Dislocations are smoothly curved without any preferred line orientation indicating no significant anisotropy in mobilities of edge and screw segments. The shear-modulus-normalized CRSS of the HEA is not exceptionally high compared to those of certain concentrated binary FCC solid solutions. Its rough magnitude calculated using the Fleischer/Labusch models corresponds to that of a hypothetical binary with the elastic constants of our HEA, solute concentrations of 20–50 at.%, and atomic size misfit of ~4%.

When the number (*n*) of constituent elements is large, the contribution of configurational entropy to the Gibbs free energy of multi-component solid solution alloys (assuming that they are ideal mixtures with random site occupancies) may be high enough to suppress compound formation and phase separation. Consequently, Yeh *et al*.^1^ defined *high-entropy alloys* (HEAs) as multi-element (*n* ≥ 5) alloys with near-equiatomic concentrations of the individual elements. Concurrently, Cantor *et al*.^2^ investigated near-equiatomic alloys containing multiple alloying elements, but referred to them as *multicomponent alloys*. Since then, this new class of alloys, whether referred to as high-entropy alloys, multicomponent alloys, multi-principal-element alloys, or compositionally complex alloys, has seen rapidly growing interest as even a cursory search of the literature shows.

Among the wide variety of HEAs that have been investigated in the last decade or so, the quinary equiatomic HEA, CrMnFeCoNi, stands out for a number of reasons and is the focus of this paper. It is known to solidify with the FCC structure^2^ and maintains its single-phase, solid-solution state at elevated temperatures^3^,^4^,^5^,^6^. Although this HEA has recently been shown to decompose into several different metallic and intermetallic phases at intermediate temperatures^4^,^5^,^6^, it can be deformation processed and recrystallized to produce single-phase equiaxed microstructures [e.g., refs 7, 8, 9, 10, 11, 12, 13]. Tests in the polycrystalline single-phase state have shown that CrMnFeCoNi exhibits certain noteworthy mechanical properties, including significant temperature dependence of yield stress coupled with moderate strain-rate sensitivity at low homologous temperatures^7^, and positive correlation between strength and ductility^7^,^8^ and strength and toughness^9^ at cryogenic temperatures. These features cannot be easily modeled in terms of the behavior of simple FCC solid solutions given that there are no “solvent” or “solute” atoms in equiatomic alloys. Although, in principle, the temperature dependence of yield stress (increasing strength with decreasing temperature) implies some sort of thermally activated process of yielding, the fundamentals of solid-solution hardening and plastic deformation have not yet been fully characterized or quantified. This is due in part to the difficulty of obtaining large single crystals of the HEA^14^. Only very recently has a paper been published on the growth and compression testing of a bulk single crystal of the CrMnFeCoNi HEA^15^; however, just one loading axis [5
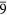
1] was tested and the authors did not perform any dislocation analysis.

As an alternative to conventional crystal growth followed by macro-scale testing, Uchic *et al*.^16^,^17^ developed a method for micro-compression testing of single-crystal pillars prepared by focused ion beam (FIB) machining. Their technique is especially useful in the case of alloys whose single crystals are difficult to prepare^17^,^18^,^19^,^20^,^21^. Here we utilize it to test micropillars of a CrMnFeCoNi HEA that were FIB machined from suitable grains of a polycrystalline sheet after grain orientations were determined by electron back scatter diffraction (EBSD). An important aspect of micropillar testing is the so-called size effect, one manifestation of which is a decrease in CRSS values with increasing pillar size, roughly following an inverse power-law relation^17^,^18^,^22^,^23^,^24^,^25^. This decrease in CRSS continues until the pillar size reaches a critical value (~20–30 μm), at which point the CRSS value obtained from micropillars may be considered as being representative of the bulk^18^,^22^,^23^,^24^,^25^. In other words, bulk CRSS values can be estimated by extrapolating the inverse power-law scaling behavior of micropillars to the critical pillar size^17^,^18^,^19^,^20^,^21^.

In the present study, we investigate the plastic deformation behavior of single crystals of the CrMnFeCoNi HEA by performing compression tests on micropillar specimens at room temperature as a function of specimen size and crystal orientation, in order to deduce its bulk CRSS value and orientation dependence. We also investigate the deformation behavior and microstructures of bulk polycrystals at room temperature and 77 K, to characterize deformation mechanisms at low homologous temperatures and deduce the stacking fault energy. Based on the results obtained, we discuss how solid-solution mechanisms contribute quantitatively to the strength of the HEA at low temperatures.

## Results

### Elastic moduli

Values of the polycrystalline elastic moduli determined at room temperature for the HEA with an average grain size of 70 μm are tabulated in Table 1, together with those previously obtained for this HEA at smaller grain sizes of 4 μm^26^ and 15 μm^10^. In the Table, the symbols *E*, *μ*, *B* and *ν* stand for Young’s modulus, shear modulus, bulk modulus, and Poisson’s ratio, respectively. For comparison, values obtained for the HEA by first-principles calculation^27^ and those for pure Ni^28^, which is the only constituent element that possesses the FCC structure, are also included. Within experimental uncertainty, the elastic moduli determined for the HEA in the present study are virtually identical to those determined in previous experimental studies (within ~2–4%), indicating that there is essentially no grain size dependence, as would be expected for randomly textured specimens in the above grain-size regime. The experimentally determined Young’s and shear moduli are also comparable to those obtained by first-principles calculation whereas the bulk modulus and Poisson’s ratio are much larger. In contrast, the experimentally determined Young’s and shear moduli and Poisson’s ratio of the HEA are ~10% smaller than those of pure Ni, whereas its bulk modulus is 24% smaller.

### Compressive stress-strain behavior of bulk polycrystalline specimens

**Figure 1:**
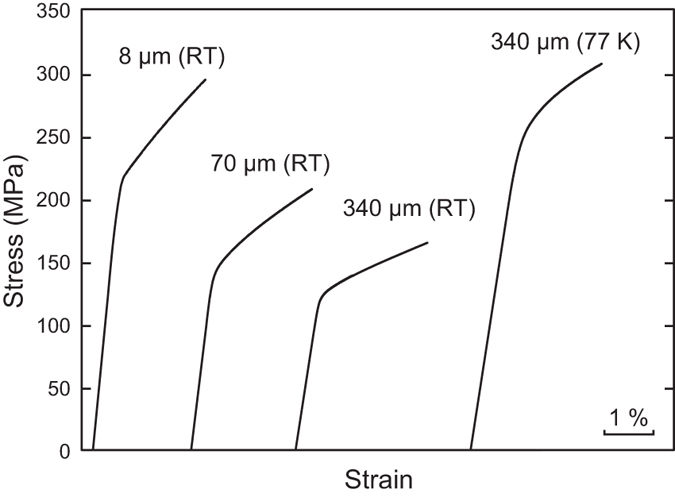
Typical compressive stress-strain curves of bulk polycrystalline specimens with different grain sizes of the equiatomic CrMnFeCoNi HEA at room temperature and 77 K.

Typical stress-strain curves of the CrMnFeCoNi HEA with different grain sizes deformed in compression at room temperature and 77 K (liquid-nitrogen temperature) are shown in Fig. 1. In all specimens, yielding and subsequent flow occurs smoothly without any discontinuities such as yield drops or serrated flow. At room temperature, the yield stress, defined here as the 0.2% offset stress increases with decreasing grain size, following conventional Hall-Petch behavior. The work-hardening rate also increases with decreasing grain size. A significant increase in yield stress occurs when the temperature is decreased to 77 K at a given grain size. For example, for 340 μm grain size, the yield stress at liquid-nitrogen temperature (260 MPa) is slightly more than twice that at room temperature (127 MPa).

### Stress-strain behavior of single-crystal micropillars

**Figure 2:**
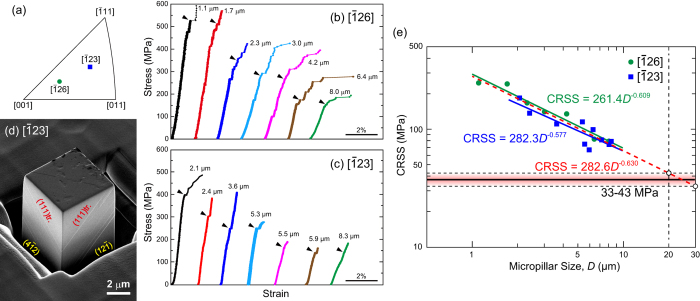
Compression tests of single-crystal micropillars of the equiatomic CrMnFeCoNi HEA. (**a**) Orientations of the loading axes of single-crystal micropillars. (**b,c**) Selected stress-strain curves of single-crystal micropillars with loading-axis orientations of (**b**) [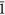
26], and (**c**) [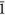
23], respectively. (**d**) Secondary-electron image taken in a scanning electron microscope showing {111} slip traces on the side surfaces of a deformed micropillar with [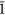
23] orientation. (**e**) Size dependence of CRSS for {111} < 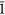
01 > slip.

Figure 2(a) shows the two loading-axis orientations ([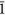
26] and [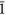
23]) tested in this study and Fig. 2(b,c) show selected stress-strain curves of micropillars with these orientations. Compression tests of some micropillars were stopped just after yielding for ease of slip line observations. Strain bursts that appear as flat regions on stress-strain curves are often observed on the stress-strain curves; they occur less frequently as the pillar size increases. These strain bursts have been interpreted as being a result of the collective motion of dislocations in an avalanche-like manner during compression of micrometer-sized single-crystalline specimens^29^,^30^. In keeping with normal practice, yield stresses were determined by the 0.2% offset method (see arrowheads in Fig. 2(b,c)). The yield stresses thus determined decrease significantly with increasing specimen size. Fig. 2(d) shows an SEM secondary electron image of a deformed micropillar soon after yielding where the viewing direction is inclined by 30° to the compression axis. Deformation markings (slip traces) observed on the two orthogonal surfaces are very straight and correspond to slip on (111).

Values of CRSS for slip on (111)[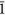
01] calculated using the yield stress values and the corresponding Schmid factors (0.488 and 0.467 for [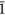
26] and [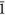
23], respectively, Table 2) are plotted in Fig. 2(e) as a function of specimen size. The CRSS values for the two orientations are virtually identical over the range of specimen sizes investigated, indicating that the CRSS value for slip on (111)[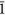
01] does not depend on crystal orientation. Therefore, it is reasonable to combine all the data points for the two orientations into one master curve with a slope of −0.63, as shown by the red dashed line in Fig. 2(e). Similar to what has been observed in single crystals of many FCC and BCC metals^17^,^18^,^22^,^23^,^24^,^25^,^31^,^32^,^33^, an inverse power-law scaling of CRSS with specimen size is also observed for the CrMnFeCoNi HEA.

### Dislocation structures

**Figure 3:**
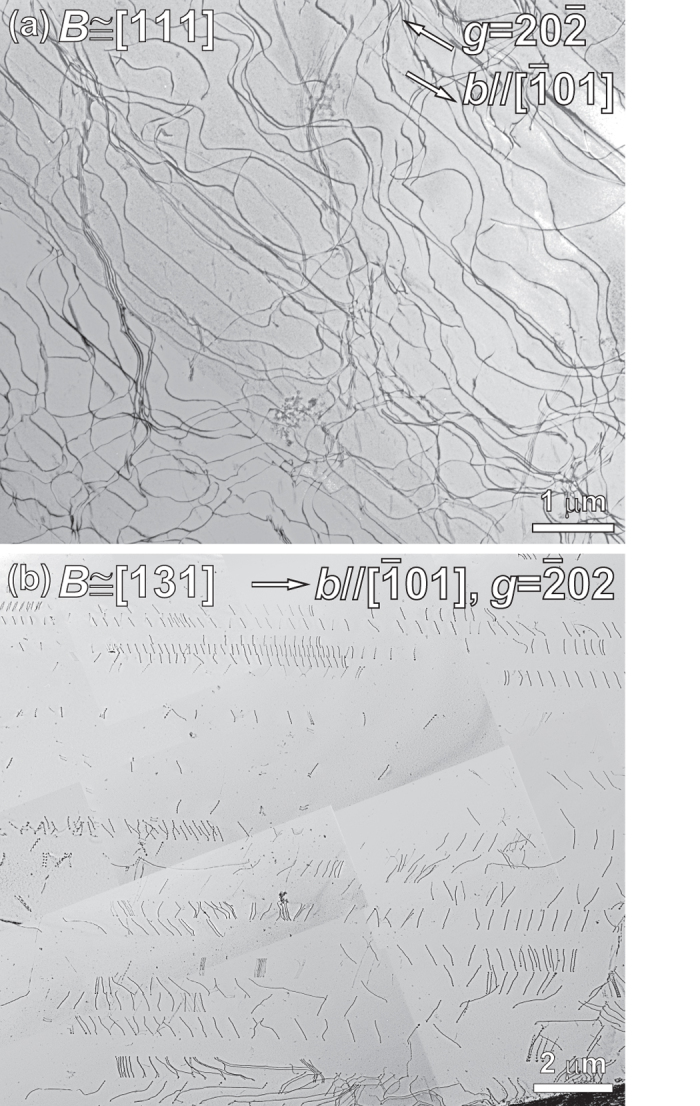
(**a**) Typical dislocation structures observed in a specimen deformed at 77 K. The thin TEM foil was cut parallel to the (111) macroscopic slip plane in a particular grain of the deformed polycrystalline specimen. (**b**) Dislocations in the form of “trains” observed in a grain adjacent to the grain shown in (**a**) with the slip planes inclined considerably from the foil surface. The reflection vectors used for imaging are indicated in each of the images.

Figure 3(a) shows a typical dislocation structure observed in a specimen deformed at liquid-nitrogen temperature. The thin foil was cut parallel to the (111) macroscopic slip plane in a particular grain of the deformed specimen. The Burgers vector (***b***) of most dislocations imaged in Fig. 3(a) was determined to be parallel to [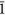
01] (not shown here). Most of these dislocations are planar and smoothly curved without any preferred line direction (i.e., they have mixed edge and screw character). The absence of any preferred orientations for the dislocations introduced during deformation at a low temperature indicates that, as in many other FCC metals, there is little anisotropy in the mobilities of the edge and screw segments. In addition, the planarity of dislocation arrangement on the slip plane suggests difficulty of cross-slip and, thus, low stacking-fault energy on {111}. This is indeed confirmed by the observation of dislocations in the form of “trains” (Fig. 3(b)) without any evidence of cross-slip in a grain adjacent to the grain of Fig. 3(a) where the slip planes are inclined considerably from the foil surface.

**Figure 4:**
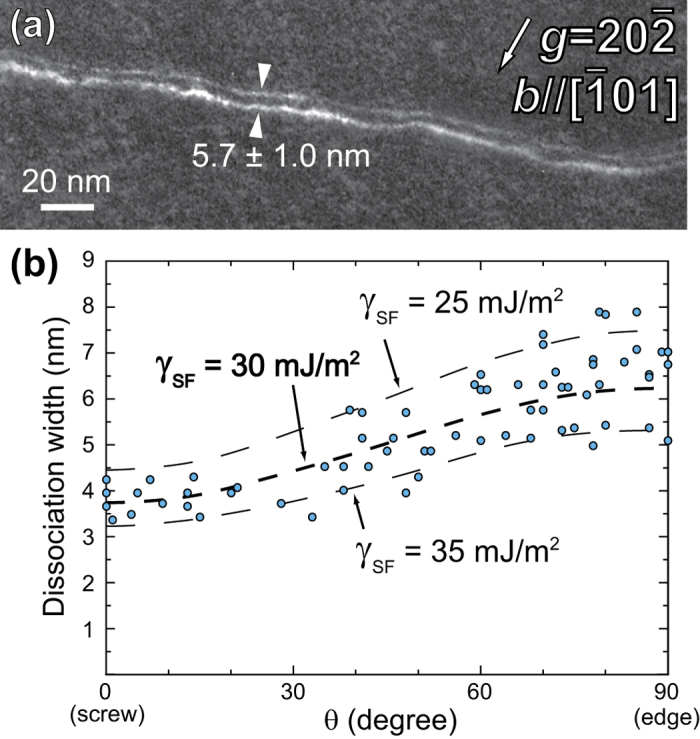
(**a**) Weak-beam TEM image of a dislocation observed in a specimen deformed at 77 K with the reflection vectors used for imaging indicated. (**b**) Corrected dissociation widths of the two Shockley partials bounding a stacking fault plotted as a function of the angle *θ* between the dislocation line direction and the total Burgers vector.

Figure 4(a) shows a typical weak-beam image of dislocations introduced during deformation at 77 K and lying on the (111) slip-plane in the grain of Fig. 3(a). The 1/2[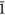
01] perfect-dislocations were confirmed to dissociate into two Shockley partials, as in many other FCC metals. The separation distance between the Shockley partials was determined to be as large as 5–8 nm for the edge orientation and somewhat smaller (~3.5–4.5 nm), as expected, for the screw. To calculate these dissociation widths, the apparent separations of the Shockley partials in the weak-beam images were corrected using the image shift correction method of Cockayne^34^ and the polycrystalline shear modulus of the HEA at 300 K (79.3 GPa, Table 1). The resulting dissociation widths of the dislocations are plotted in Fig. 4(b) as a function of the angle *θ* between the dislocation line direction and the total Burgers vector. Comparable dissociation widths were recently measured by high-resolution TEM in this HEA^35^. The three dashed curves in Fig. 4(b) are derived from the DISDI program^36^, which is based on Stroh’s formalism for anisotropic elasticity^37^. From the plot of Fig. 4(b), the stacking fault energy (*γ*_SF_) is estimated to be 30 ± 5 mJ/m^2^, which is slightly higher than that estimated for the HEA by *ab initio* calculations (18.3–27.3 mJ/m^2^)^27^. Our experimentally determined stacking fault energy is relatively low compared to those of pure FCC metals (see Table 3) but not that low when compared to certain FCC alloys, such as the Cu-Al binaries listed in Table 3. Here it is important to note that, although the separation distance between the Shockley partials can be relatively large (5–8 nm), the stacking fault energy of the HEA is not particularly low. This is because of its relatively high shear modulus. In fact, when the separation distance between partials (*r*) is normalized by the magnitude of the Burgers vector (*b*) of the relevant perfect dislocation, the value of *r*/*b* for the HEA is much greater than that of pure FCC metals (Table 3). This is consistent with our observation (Fig. 3(a,b)) that cross-slip of dislocations is difficult in the HEA.

## Discussion

Inverse power-law scaling of CRSS with specimen size has been reported for many single-crystal micropillars of FCC and BCC metals, e.g., refs 18,22 and 33. The power-law slope is usually in the range of –0.5 to –1.0 for FCC metals^17^,^18^,^22^,^23^,^24^,^25^,^31^, and in the range of –0.2 to –0.5 for BCC metals^32^,^33^. For the present HEA, the power-law slope obtained by fitting all the data points for the two orientations is –0.630 (Fig. 2(e)), which is within the range reported for FCC metals but close to the lower bound. This is reasonable given the high CRSS value of the HEA compared to those of pure FCC metals (as discussed later), and in view of the interpretation^38^ that the flow stress of micropillars consists of three terms: (i) the friction stress term corresponding to the bulk yield stress, (ii) the elastic interaction term resulting from dislocation-dislocation interaction given by the Bailey-Hirsch type equation and (iii) the line tension term for the operation of single-arm dislocation sources, giving rise to size-dependent strength. Consequently, the magnitude of the power-law slope of CRSS (yield stress) attributable to (iii) decreases with increasing friction stress (i.e., a higher friction stress tends to decrease the slope).

For many different FCC metals the bulk CRSS value can be deduced from the micropillar results by extrapolating the obtained power-law plot of the CRSS versus specimen size to a specimen size of 20–30 μm^23^,^29^. This was first reported for Ni^23^,^29^ and subsequently for Cu^31^, Au^39^ and so on. The physical basis for this critical size of 20–30 μm may be understood as follows. The lengths of double-ended Frank-Read sources operative in bulk FCC metals at the CRSS level are 10^4^ to 10^5^ times the Burgers vector, or approximately 10 μm. Assuming that the transition to bulk-like behavior occurs when the dislocation multiplication source changes from single-arm to double-ended Frank-Read source, the pillar size that is representative of the bulk should be two to three times the length of the double-ended Frank-Read source (i.e., 20–30 μm). Using this extrapolation method, the bulk CRSS value for slip on {111} < 110 > of the HEA is estimated to be 33–43 MPa, which is much greater than those of pure FCC metals (for example, it is only 3.2 MPa for Ni^40^), giving rise to the low power-law slope of CRSS (Fig. 2(e)). Recently, we have succeeded in growing single crystals of the CrMnFeCoNi HEA and our preliminary study has confirmed that at room temperature, these single crystals exhibit CRSS values of around 45 MPa, which conforms to the bulk CRSS values estimated from the present micropillar compression tests. The full results of deformation experiments on bulk single crystals of the HEA will be published elsewhere. In contrast to the above, Patriarca *et al*.^15^ reported a much higher CRSS value of 70 MPa at room temperature for bulk single crystals. Their value is also significantly higher than what can be deduced from the polycrystalline yield stress using the Taylor factor (3.06). For example, the uniaxial yield stress of polycrystalline CrMnFeCoNi is ~150–160 MPa for 155-μm grain size^8^, which implies a CRSS of ~49–52 MPa. This derived CRSS value of ~50 MPa should actually be considered an *upper limit* since Hall-Petch strengthening is known to affect the polycrystalline yield strength of this alloy as discussed below. Impurity contamination during crystal growth may be a contributory factor to the higher CRSS reported in ref. 15, but additional studies are needed to improve understanding.

The perfect dislocation in the CrMnFeCoNi HEA is observed to dissociate into two Shockley partial dislocations with a stacking fault in between. The absence of any significant orientation dependence of CRSS is consistent with this dissociation mode, since atomistic simulations by Vitek and co-workers^41^,^42^ have indicated that the core spreading is always planar for Shockley partial dislocations in FCC metals. The stacking fault energy (30 ± 5 mJ/m^2^) of the HEA deduced from the separation distance of the two, coupled, Shockley partial dislocations is not dramatically lower than that of certain FCC metals (Table 3). This is due to the relatively high shear modulus of the HEA. Indeed, when the dissociation width is normalized by the magnitude of the perfect Burgers vector (*r*/*b*), the normalized width is much larger than those for pure FCC metals (Table 3). This may account for the difficulty in cross-slip as observed in the present study and for the occurrence of deformation twinning at low temperatures as observed by Otto *et al*.^8^.

**Figure 5:**
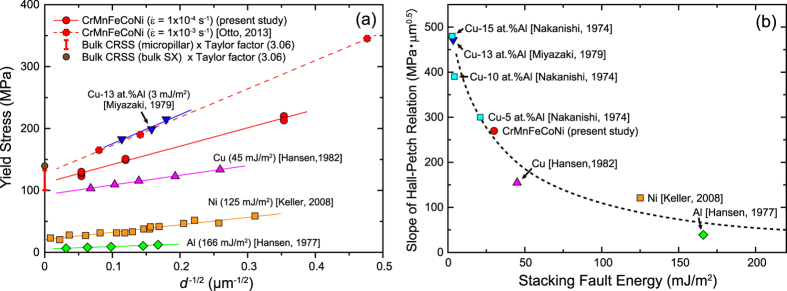
(**a**) Hall-Petch plot of the grain size dependence of yield stress for the polycrystalline HEA at room temperature at a strain rate of 1 × 10^−4^ s^−1^ (this study), together with data for polycrystals of the same HEA tested previously at a strain rate of 1 × 10^−3^ s^−1^ by Otto *et al*.^8^ and for some other FCC metals and alloys. (**b**) Comparison of the Hall-Petch slopes of the HEA and some other FCC metals and alloys as a function of stacking fault energy.

Figure 5(a) shows a Hall-Petch plot of the compressive yield stress of the polycrystalline HEA at room temperature at a strain rate of 1 × 10^−4^ s^−1^. The figure also includes data for polycrystals of the same HEA tensile tested at a strain rate of 1 × 10^−3^ s^−1^ by Otto *et al*.^8^ and for some other FCC metals and alloys for comparison^43^,^44^,^45^,^46^. The bulk CRSS value estimated in the present micropillar compression study for the HEA is plotted as the yield stress value at *d*^−1/2^ = 0 after multiplying by the Taylor factor (3.06). The Hall-Petch relation is observed to be valid for the HEA including the yield stress value at *d*^−1/2^ = 0. The Hall-Petch slope for the HEA tested at a strain rate of 1 × 10^−4^ s^−1^ in the present study is lower than that obtained for the same HEA tested at a strain rate of 1 × 10^−3^ s^−1^ by Otto *et al*.^8^. This may be due to the different strain rates employed. In general, the Hall-Petch slope of FCC metals and alloys is known to increase with decreasing stacking fault energy, as seen in Fig. 5(a,b). The observed Hall-Petch slope for the HEA is comparable to that for Cu-5at.%Al alloys^47^. Although the stacking fault energy (21 mJ/m^2^) of Cu-5at.%Al^48^ is a bit lower than that of the HEA, their normalized separation distances (*r*/*b*) are comparable (Table 3). This is clear evidence for the validity of the HEA stacking-fault energy determined in the present study.

**Figure 6:**
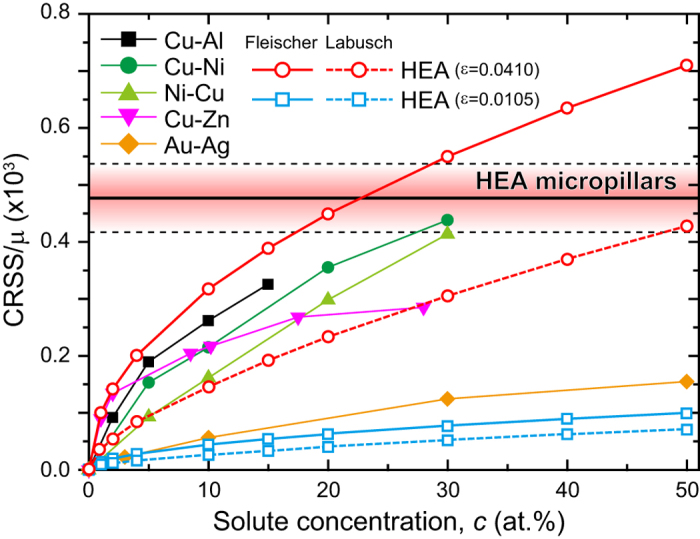
CRSS values (33–43 MPa) determined by micropillar compression tests are normalized by the shear modulus and plotted as a band independent of the solute concentration. The extent of solid-solution hardening in the equiatomic HEA roughly estimated by a simplified application of the classical Fleischer model^49^ and modified Labusch model^50^,^51^ for two different atomic size misfits is plotted as a function of “solute” concentration (see text). For comparison, the CRSS values normalized by the corresponding shear moduli of some binary Cu, Ni and Au FCC solid solutions are also plotted as a function of solute concentration.

The deformation of the HEA is not related to differential mobilities of edge and screw dislocations, as is evident from the absence of preferred orientations for dislocations introduced during deformation at low temperatures (Fig. 3). Rather, the strong negative temperature dependence of yield stress observed for the HEA (Fig. 1) is due to the thermal component of solid-solution hardening. We now consider how the HEA is strengthened through solid-solution hardening. For comparison, the experimentally deduced CRSS values at room temperature normalized by the corresponding shear modulus of certain binary FCC solid solutions of Cu, Ni and Au alloys are plotted in Fig. 6 as a function of solute concentration. The extent of solid solution hardening increases with increase in solute concentration for all the alloys listed in Fig. 6, but the effect is more pronounced as the atomic size misfit between the solvent and solute atoms increases. For the HEA, the CRSS determined in the present study (33–43 MPa) normalized by its shear modulus is plotted in Fig. 6 as a band independent of the solute concentration. When the normalized CRSS of the HEA is compared to those of the binary alloys, it is seen that it is higher than anything achievable by the Cu-Zn and Au-Ag binaries at any concentration but comparable to those of the Cu-30 at.% Ni and Ni-30 at.% Cu alloys.

We now make a rough estimate of the extent of solid-solution hardening of the equiatomic HEA by simply adopting the classical Fleischer model^49^ and modified Labusch model^50^,^51^, which were originally applied to dilute and relatively concentrated binary solid-solutions, respectively (details are shown in the supplementary information). The above treatment is based on the assumption that atomic size misfit makes a major contribution to solid solution hardening and the contribution of the shear modulus misfit can be neglected. This is believed to be a reasonable approximation in many FCC solid solutions^52^,^53^. Although the concentration (20 at. %) of each constituent element in the HEA is higher than that in typical dilute solid solutions, and there is no clear way to differentiate between “solvent” and “solute” atoms in an equiatomic alloy, we make a rough estimate by assuming that the atoms with the largest and smallest sizes are the “solvent” and “solute” atoms, respectively. As tabulated in Table 4, Co and Mn have, respectively, the largest and smallest Goldschmidt radii^54^, which results in an atomic size (radius) misfit of 1.05% for this pair. The extent of solid solution hardening in the HEA can then be calculated by assuming the Fleischer and modified Labusch models^49^,^50^,^51^ as a function of solute concentration for a series of hypothetical Co-Mn binary alloys and the results are plotted in Fig. 6. Clearly, for all concentrations the strength of the “equivalent” HEA calculated in this way is far lower than the experimentally determined strength. This may be due to the fact that the Goldschmidt radii do not give accurate values of the size misfit in the alloy. When the atomic radii of the constituent atoms in the quinary equiatomic HEA are estimated by *ab initio* calculations (details are shown in the supplementary information), the largest atomic misfit is found to be 4.1% between Cr and Co (or Fe), as tabulated in Table 4. For this misfit value, the extent of solid solution hardening expected in the HEA can be calculated as a function of solute concentration for a series of hypothetical Co-Cr binary alloys and those results are also plotted in Fig. 6. The normalized CRSS value determined from the HEA micropillar experiments is similar to that of the hypothetical Co-Cr binary alloys at solute concentrations of approximately 20 at.% and 50 at.% for the Fleischer and modified Labusch models, respectively. Although this is just an order-of-magnitude estimate of solid-solution hardening in the HEA, it shows that the strength of the HEA is not exceptionally high but is in the expected range for FCC alloys, including certain highly concentrated binaries, provided that there is an atomic radius mismatch of ~4%. It thus provides a first estimate of the magnitude of the often cited “severe” lattice distortions needed to rationalize the significantly (10×) higher CRSS of the HEA compared to those of pure FCC metals.

## Conclusions

1. By room-temperature compression of single-crystal micropillars of the CrMnFeCoNi HEA, we have shown that the CRSS for slip on {111} < 110 > follows inverse power-law scaling with size, independent of crystal orientation. The power-law slope is –0.63, which is near the lower bound of values reported in the literature for FCC metals (–0.5 to –1.0).
2. Extrapolation of the size-dependent CRSS to a critical pillar size of 20–30 μm yields a value of 33–43 MPa for the bulk CRSS. This bulk CRSS is much higher than those of pure FCC metals (for example, it is only ~3 MPa for Ni), giving rise to its weaker size dependence compared to pure FCC metals.
3. However, the bulk CRSS of the CrMnFeCoNi HEA is not exceptionally high when compared with those of certain FCC solid solution alloys. In fact, its magnitude can be roughly calculated using simple Fleischer and Labusch models for a binary alloy having solute concentrations of 20–50 at.%, a size misfit of ~4%, and the elastic constants of our HEA.
4. The Young’s and shear moduli and Poisson’s ratio of the CrMnFeCoNi HEA are smaller by ~10% than those of pure Ni, whereas its bulk modulus is smaller than that of Ni by as much as 24%.
5. The yield stress of the CrMnFeCoNi HEA increases significantly as the temperature is decreased from room temperature to 77 K. The strong negative temperature dependence of yield stress is due to the thermal component of solid-solution hardening and not due to significantly different mobilities of the edge and screw dislocation segments, as is evident from the absence of preferred orientations for dislocations introduced during deformation at low temperatures. This behavior is similar to that of typical FCC metals and different from that of many BCC metals.
6. Most dislocations are confined to their original slip planes forming a planar arrangement without significant cross-slip at low strains. The perfect dislocation in the HEA is observed to dissociate into two Shockley partial dislocations with a stacking fault in between. The stacking fault energy of the HEA deduced from the separation distance of the two, coupled, Shockley partial dislocations is 30 ± 5 mJ/m^2^, which is relatively low but not particularly so when compared with certain FCC metals and alloys. Although the stacking fault energy is not particularly low, the separation distance between the Shockley partials can be large (up to 8 nm) (due to its relatively high shear modulus), accounting for the difficulty in cross-slip and for the occurrence of deformation twinning at low temperatures.
7. The Hall-Petch relation is observed to be valid for the HEA with a relatively high Hall-Petch slope, consistent with the relatively low stacking-fault energy. The observed Hall-Petch slope for the HEA is comparable to that of Cu-5at.%Al alloys with a similar dissociation width for the two coupled Shockley partial dislocations.

## Methods

High-purity (>99.9%) Cr, Mn, Fe, Co and Ni in equiatomic concentrations were arc-melted in argon atmosphere and drop-cast into a cylindrical copper mold. The drop-cast bar was cold-rolled to a total thickness reduction of 60% without intermediate annealing and portions were recrystallized at 1,173, 1,373, or 1,473 K for 1, 3 and 168 h, respectively. The recrystallized microstructures were almost equi-axed with an average grain size of approximately 8, 70 and 340 μm, respectively, for the three different heat treatments above. A rectangular parallelepiped specimen with dimensions of approximately 3 × 3 × 3 mm^3^ was cut by spark-machining and was mechanically polished with SiC paper and then with diamond paste. Measurements of polycrystalline elastic moduli were carried out by the resonance ultrasound spectroscopy (RUS) method^55^ at room temperature. After mechanical and electrolytic polishing, single-crystal micropillar specimens with square cross-sections having aspect ratios of ~3:1 (height to edge length) were machined from the recrystallized grains with a focused-ion beam (FIB) apparatus at an operating voltage of 30 kV. The edge lengths of the square cross-sections (referred to as “specimen size” in this paper) ranged from 1.0 to 8.3 μm. The compression axis orientations of these micropillar specimens as well as their side-face orientations were examined by electron backscatter diffraction (EBSD) in a field-emission scanning electron microscope (FE-SEM). Two different compression axis orientations ([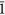
23] and [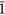
26]) (Fig. 2(a)) were investigated in order to see if the critical resolved shear stress (CRSS) for slip on the (111)[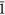
01] primary slip system depends on crystal orientation. As listed in Table 2, the Schmid factors for (111)[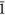
01] slip are almost identical for the two orientations, whereas the *Q* parameter defined by the Schmid factor ratio (111)[1
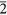
1]/(111)[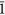
01]^56^ is positive ( + 0.082) and negative (–0.289) for the [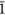
26] and [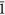
23] orientations, respectively. In L1_2_ intermetallic compounds based on the FCC lattice, the *Q* parameter has been shown to give rise to the orientation dependence of CRSS for slip on (111)[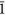
01] through the constriction process of two coupled Shockley partials^41^. Compression tests were conducted on the micropillar specimens with a flat punch indenter tip mounted in a nano-indentation tester at room temperature and at a constant strain rate of 1 × 10^−1^ s^−1^. In addition, compression tests on bulk polycrystalline specimens with dimensions of 2 × 2 × 6 mm^3^ were also conducted on an Instron-type testing machine at 77 K and room temperature at a strain rate of 1 × 10^−4^ s^−1^ up to a plastic strain of 1%. Dislocation structures were examined with a JEM-2000FX transmission electron microscope (TEM) operated at 200 kV. Thin foils for TEM observations were prepared from the deformed bulk specimens by cutting slices of about 100 μm in thickness at an angle 45° inclined from the compression axis orientation. These slices were subjected to twin-jet electro-polishing for perforation in a solution of perchloric acid, nitric acid and methanol (2:1:9, v/v) at 233 K and 6 V.

## Additional Information

**How to cite this article**: Okamoto, N. L. *et al*. Size effect, critical resolved shear stress, stacking fault energy, and solid solution strengthening in the CrMnFeCoNi high-entropy alloy. *Sci. Rep.* **6**, 35863; doi: 10.1038/srep35863 (2016).

## Supplementary Material

Supplementary Information

## Figures and Tables

**Table 1:** Polycrystalline elastic moduli of the equiatomic CrMnFeCoNi HEA measured by the RUS method and first principles calculation together with those for pure Ni.

	*E* (GPa)	*μ* (GPa)	*B* (GPa)	*ν*
CrMnFeCoNi HEA (300 K)^a^	200.3	79.3	140.8	0.263
CrMnFeCoNi HEA (300 K)^b^	202	80	143	0.265
CrMnFeCoNi HEA (300 K)^c^	203	81	137	0.253
CrMnFeCoNi HEA (0 K)^d^	207	86	117	0.204
Ni (300 K)^e^	223	86	184	0.296

^a^Present study.

^b^Haglund *et al*.^26^.

^c^Laplanche *et al*.^10^.

^d^Zaddach *et al*.^27^.

^e^Alers *et al*.^28^.

**Table 2:** Schmid factors and *Q* parameters for compression axes of [ 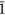 26] and [ 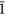 23].

	Schmid factor (111)[ 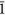 01]	Schmid factor (111)[1 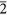 1]	*Q* parameter
[ 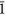 26]	0.488	−0.040	+ 0.082
[ 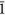 23]	0.467	0.135	−0.289

**Table 3:** Stacking fault energy (γ_SF_), magnitude of Burgers vector (*b*) for a perfect dislocation, dislocation dissociation width (*r*) normalized by the magnitude of Burgers vector, and polycrystalline shear modulus (*μ*) for the equiatomic CrMnFeCoNi HEA, pure Cu, Ni, Al and Cu-Al alloys.

	γ_SF_ (mJ/m^2^)	*b*_perfect_ (nm)	*r*/*b*_perfect_		*μ* (GPa)
			screw	edge	
CrMnFeCoNi HEA	30^a^	0.255	14.7	24.3	79.3^a^
Cu	45^b^	0.255	5.2	12.9	47^f^
Ni	125^c^	0.249	3.6	7.5	86^f^
Al	166^d^	0.286	0.9	2.2	26^f^
Cu-5Al	21.1^e^	0.255	11.1	26.7	46^f^
Cu-10Al	4.2^e^	0.255	55.8	133	46^f^
Cu-15Al	2.8^e^	0.255	83.7	197	45^f^

^a^Present study.

^b^C.B. Carter & I.L.F. Ray^57^.

^c^C.B. Carter & S.M. Holmes^58^.

^d^L.E. Murr^59^.

^e^A. Howie & P.R. Swann^48^.

^f^G. Simmons^60^.

**Table 4:** Goldschmidt^54^ and effective atomic radii of the constituent elements.

(pm)	Cr	Mn	Fe	Co	Ni
Goldschmidt radius	124.9	124.0	124.1	125.3	124.6
Effective atomic radius	126.9	123.5	121.9	121.9	123.9

The effective atomic radii were derived by *ab initio* calculations (details are shown in the supplementary information).
