# Supplementary material for: Size effect, critical resolved shear stress, stacking fault energy, and solid solution strengthening in the CrMnFeCoNi high-entropy alloy
Source: Sci Rep. 2016 Oct 24;6:35863. doi: 10.1038/srep35863 (PMC5075899; doi:10.1038/srep35863)
Supplement: Supplementary Information [file srep35863-s1.pdf]

# Supplementary Information:

## Size effect, critical resolved shear stress, stacking fault energy, and solid solution strengthening in the CrMnFeCoNi high-entropy alloy

Norihiko L. Okamoto[1,2,*], Shu Fujimoto[1], Yuki Kambara[1], Marino Kawamura[1], Zhenghao M. T. Chen[1], Hirotaka Matsunoshita[1], Katsushi Tanaka[3], Haruyuki Inui[1,2], and Easo P. George[4,5]

[1]Department of Materials Science and Engineering, Kyoto University, Kyoto 606-8501, Japan
[2]Center for Elements Strategy Initiative for Structure Materials (ESISM), Kyoto University, Kyoto 606-8501, Japan
[3]Department of Mechanical Engineering, Kobe University, Nada-ku, Kobe 657-8501, Japan
[4]Materials Science and Technology Division, Oak Ridge National Laboratory, Oak Ridge, TN 37831, USA
[5]Present address: Institute for Materials, Ruhr University Bochum, Universitätsstr. 150, Bochum 44801, Germany
*Corresponding author, email: okamoto.norihiko.7z@kyoto-u.ac.jp

**Expressions of the classical Fleischer model and modified Labusch model**

In the classical Fleischer model[1], which was originally applied to dilute binary solid solutions, the extent of solid solution hardening ($\tau$) is expressed with the following equations,

$$\tau = \frac{F_{\mathrm{max}}^{3/2}}{b^3}\left(\frac{c}{\mu}\right)^{1/2} \tag{1}$$

$$\left|F_{\mathrm{max}}\right| = \frac{\sqrt{3}\mu b}{2h^2}\left(\frac{1+\nu}{1-\nu}\right)\varepsilon r_{\mathrm{m}}^3 \tag{2}$$

$$\varepsilon = \frac{r_{\mathrm{s}} - r_{\mathrm{m}}}{r_{\mathrm{m}}} \quad (r_{\mathrm{m}}\text{: atomic radius of solvent, } r_{\mathrm{s}}\text{: atomic radius of solute}) \tag{3}$$

where $c$, $b$, $\mu$, $\nu$ and $h$ stand for the solute concentration, magnitude of the perfect Burgers vector, shear modulus, Poisson's ratio, and interplanar distance between slip planes, respectively. $F_{\mathrm{max}}$ is the miximum interaction force between a dislocation and a solute whereas $\varepsilon$ is the atomic size misfit between solvent (matrix) and solute atoms.

The original Labusch model[2], which statistically treats the interaction between a dislocation and solute atoms with various interaction forces, is better suited to concentrated binary solid solutions than the Fleischer model. According to the Labusch model[2], the extent of solid solution hardening is proportional to the two-thirds power of the solute concentration ($c^{2/3}$ dependence) in contrast to the $c^{1/2}$ dependence in the Fleischer model. Since the estimation of absolute values of solid solution hardening with the Labusch model is laborious and needs

Green functions, we adopted instead a modified Labusch model[3], in which the same interaction statistics as in the original Labusch model are implemented. In the modified Labusch model, the extent of solid solution hardening is expressed with the following equations[3],

$$\tau = 1.26\mu\left(\frac{w}{b}\right)^{1/3} c^{2/3}\overline{\hat{K}}^{4/3} \tag{4}$$

$$\overline{\hat{K}} = \frac{1}{2}\left(\hat{K}_{edge} + \hat{K}_{screw}\right) \approx \frac{1}{2}\left(-0.181\varepsilon + 0.041B\varepsilon\right), \tag{5}$$

where $w$, $B$, $\hat{K}_{edge}$, $\hat{K}_{screw}$ and $\overline{\hat{K}}$ stand for the range of solute interaction, a constant (between 0.3 and 1.0), the normalized resistive forces for the edge and screw dislocations and their arithmetic mean, respectively. In the present study, $w$ and $B$ are assumed to be three times the (111) interplanar distance and unity, respectively.

**Derivation of the effective atomic radii in the quinary equiatomic HEA**

In order to estimate the atomic radius for each of the constituent elements in the quinary HEA based on *ab initio* calculations, we constructed special quasi-random structures (SQSs)[4] representing perfectly random structures of five different quaternary alloys with different combinations of four of the five elements (MnFeCoNi, CrFeCoNi, CrMnCoNi, CrMnFeNi and CrMnFeCo). We eventually obtained SQSs containing 256 atoms of 4×4×4 extension of FCC unit cell in which linearly-independent 24 correlations from first to fourth nearest-neighbour pairs in the FCC lattice were optimized[5]. The supercell volume, cell shape and atomic positions were relaxed by first-principles total-energy calculations based on density functional theory. We employed VASP code[6] based on the projector-augmented wave method within the generalized-gradient approximation of Perdew-Burke-Ernzerhof to the exchange-correlation functional. The plane-wave cutoff of 350 eV was used. The supercells were relaxed until the residual forces became less than $10^{-3}$ eV/angstrom. An average atomic radius of the four constituent elements in each of the quaternary SQSs was derived from the relaxed supercell volumes by assuming the rigid sphere model, and then, the effective atomic radii of the five elements in the quinary HEA were deduced by applying a maximum likelihood method to minimize the difference between the relaxed and estimated supercell volumes.

**References**

1. Fleischer, R. L. Substitutional solution hardening. *Acta Metall.* **11**, 203-209 (1963).
2. Labusch, R. A Statistical Theory of Solid Solution Hardening. *Phys. Status Solidi* **41**, 659-

669 (1970).

3. Argon, A. S. *Strengthening mechanisms in crystal plasticity*. Ch. 5, 136-192 (Oxford University Press, 2008).
4. Zunger, A., Wei, S. H., Ferreira, L. G. & Bernard, J. E. Special Quasirandom Structures. *Phys. Rev. Lett.* **65**, 353-356 (1990).
5. Yuge, K. Estimation of Macroscopic Physical Property in Disordered States: Special Microscopic States Approach. *J. Phys. Soc. Jpn.* **84**, 084801 (2015).
6. Kresse, G. & Furthmuller, J. Efficient iterative schemes for ab initio total-energy calculations using a plane-wave basis set. *Phys. Rev. B* **54**, 11169-11186 (1996).
